# Transcriptome-Wide Expression Profiling in Skin Fibroblasts of Patients with Joint Hypermobility Syndrome/Ehlers-Danlos Syndrome Hypermobility Type

**DOI:** 10.1371/journal.pone.0161347

**Published:** 2016-08-12

**Authors:** Nicola Chiarelli, Giulia Carini, Nicoletta Zoppi, Chiara Dordoni, Marco Ritelli, Marina Venturini, Marco Castori, Marina Colombi

**Affiliations:** 1 Department of Molecular and Translational Medicine, Division of Biology and Genetics, University of Brescia, Bres6cia, Italy; 2 Department of Clinical and Experimental Sciences, Division of Dermatology, Spedali Civili University Hospital, Brescia, Italy; 3 Department of Molecular Medicine, Unit of Medical Genetics, Sapienza University, San Camillo-Forlanini Hospital, Rome, Italy; Ecole normale superieure de Lyon, FRANCE

## Abstract

Joint hypermobility syndrome/Ehlers–Danlos syndrome hypermobility type (JHS/EDS-HT), is likely the most common systemic heritable connective tissue disorder, and is mostly recognized by generalized joint hypermobility, joint instability complications, minor skin changes and a wide range of satellite features. JHS/EDS-HT is considered an autosomal dominant trait but is still without a defined molecular basis. The absence of (a) causative gene(s) for JHS/EDS-HT is likely attributable to marked genetic heterogeneity and/or interaction of multiple loci. In order to help in deciphering such a complex molecular background, we carried out a comprehensive immunofluorescence analysis and gene expression profiling in cultured skin fibroblasts from five women affected with JHS/EDS-HT. Protein study revealed disarray of several matrix structural components such as fibrillins, tenascins, elastin, collagens, fibronectin, and their integrin receptors. Transcriptome analysis indicated perturbation of different signaling cascades that are required for homeostatic regulation either during development or in adult tissues as well as altered expression of several genes involved in maintenance of extracellular matrix architecture and homeostasis (e.g., *SPON2*, *TGM2*, *MMP16*, *GPC4*, *SULF1*), cell-cell adhesion (e.g., *CDH2*, *CHD10*, *PCDH9*, *CLDN11*, *FLG*, *DSP*), immune/inflammatory/pain responses (e.g., *CFD*, *AQP9*, *COLEC12*, *KCNQ5*, *PRLR*), and essential for redox balance (e.g., *ADH1C*, *AKR1C2*, *AKR1C3*, *MAOB*, *GSTM5*). Our findings provide a picture of the gene expression profile and dysregulated pathways in JHS/EDS-HT skin fibroblasts that correlate well with the systemic phenotype of the patients.

## Introduction

Ehlers-Danlos syndromes (EDS) are a heterogeneous group of heritable connective tissue disorders (HCTDs) sharing a variable combination of skin hyperextensibility, internal organ and vessel fragility and dysfunctions, and generalized joint hypermobility (gJHM) [1, 2]. Six major EDS types are recognized by specific diagnostic criteria in the Villefranche nosology [3]. Among them, the EDS hypermobility type (EDS-HT, OMIM#130020) is likely the most common [4]. The genetic basis of EDS-HT is still unknown. Hence, EDS-HT is an exclusion diagnosis in presence of gJHM, joint instability complications, smooth, velvety, and/or mildly hyperextensible skin, and positive family history. EDS-HT is considered clinically undistinguishable from the joint hypermobility syndrome (JHS), which was originally recognized by the Brighton criteria [5]. Expert opinion and segregation studies confirm such a clinical impression [6, 7] and the identification of a unified set of diagnostic criteria is underway.

The actual difficulties in recognizing JHS/EDS-HT are due to the low specificity of available diagnostic criteria and the lack of any confirmatory test. Clinical variability is wide and now includes functional gastrointestinal disorders, cardiovascular dysautonomia, and gynecological manifestations, all not comprised in the available diagnostic criteria [8–10]. While tradition defines JHS/EDS-HT as an autosomal dominant trait, incomplete penetrance, variable expressivity and markedly skewed sex ratio lead to hypothesize a much more complex molecular basis for JHS/EDS-HT. The recent identification of a locus on chromosome 8 linked to JHS/EDS-HT in a Belgian multiplex family supports the existence of major Mendelian factors at least in selected families [11]. However, marker locus heterogeneity and complex (i.e., non-Mendelian) inheritance patterns are valid hypotheses that need to be explored in depth.

To gain insights into the pathogenesis of JHS/EDS-HT, we performed a transcriptome-wide expression profiling in five skin fibroblast strains, derived from adult patients with full-blown characteristics and which showed a common disarray of several extracellular matrix (ECM) structural proteins. Our work adds insights into etiopathogenesis of JHS/EDS-HT for future studies aimed at deciphering the molecular basis of such a protean disorder.

## Materials and Methods

### Clinical evaluation of JHS/EDS-HT patients

This study was approved by the local Ethical Committee “Comitato Etico Provincia di Brescia, ASST Spedali Civili, Brescia, Italia”, registration number NP2378 and performed in accordance with the Declaration of Helsinki Principles. Written informed consent was obtained from all patients and controls to the study and for skin biopsy. Patients selected for this study were clinically evaluated in the Centre of Heritable Connective tissue disorders and Ehlers-Danlos syndromes of the Spedali Civili of Brescia. Criteria included in the Villefranche nosology for EDS-HT and Brighton criteria for JHS were used for assessing JHS/EDS-HT patients. gJHM was evaluated using the Beighton score (BS); patients with negative BS (≤ 5/9) were investigated for historical JHM using the 5-point questionnaire [12]. Intensity of chronic pain was evaluated using the numerical rating scale (NRS-11). The presence/absence of some additional recurrent findings not included in the above mentioned sets of diagnostic criteria, i.e., chronic fatigue, functional gastrointestinal disorders, dysautonomia, atopy, and neuropathic pain, was also annotated. For this study, we recruited 5 adult female patients (P1-P5) with a comparably full-blown phenotype.

### Cell cultures and antibodies

Skin fibroblast cultures from five JHS/EDS-HT female patients, four positive both for the Villefranche and Brighton criteria and one for the Brighton criteria, and six unrelated sex-matched healthy donors were established in our lab from skin biopsies by standard protocols. Controls had a negative BS and did match neither the Villefranche nor the Brighton criteria, as well as those for the other HCTDs. Dermal fibroblasts were grown *in vitro* at 37° C in a 5% CO_2_ atmosphere in Earle’s Modified Eagle Medium (MEM) supplemented with 2 mM L-glutamine, 10% FBS, 100 μg/ml penicillin and streptomycin (Life Technologies, Carlsbad, CA, USA). Fibroblasts were expanded until full confluency and then harvested by 0.25% trypsin/0.02% EDTA treatment at the same passage number (from 3rd to 4th).

Goat anti-type I collagen (COLLI) polyclonal antibody (Ab), rabbit anti-type III collagen (COLLIII) Ab, mouse anti-elastin (ELN) (clone 10B8), anti-α5β1 (clone JBS5), anti-αvβ3 (clone LM609), and anti-α2β1 (clone BHA.2) integrin monoclonal antibodies (mAbs) were from Millipore-Chemicon Int. (Billerica, MA). Goat anti-type V collagen (COLLV) Ab was purchased from LifeSpan BioSciences, Inc. (Seattle, WA). Anti-fibrillins (FBNs) (clone 11C1.3) mAb was from NeoMarkers (Fremont, CA). The rabbit Ab against human fibronectin (FN) and mAb against all of the human isoforms of tenascin (TNs) (clone BC-24) were from Sigma Chemicals (St. Louis, MO). Rhodamine-conjugated anti-goat secondary Ab was obtained from Calbiochem-Novabiochem INTL, Alexa Fluor 488 anti-rabbit and Alexa Fluor 594 anti-mouse were from Life Technologies.

### Immunofluorescence microscopy (IF)

To analyze the FN, COLLI, COLLIII, COLLV, and TNs ECM organization, JHS/EDS-HT fibroblasts were immunoreacted as described previously [13, 14]. In brief, cold methanol fixed fibroblasts were immunoreacted with 1:100 anti-FN, anti-COLLV, anti-COLLIII, anti-COLLI Abs, or with 1 μg/ml anti-TNs mAb. For analysis of α2β1, α5β1, and αvβ3 integrins, cells were fixed in 3% PFA/60 mM sucrose and permeabilized in 0.5% Triton X-100 as reported in detail previously [13]. In particular, controls and JHS/HT-EDS fibroblasts were reacted for 1 h at room temperature with 4 μg/ml anti-α5β1, anti-αvβ3, and anti-α2β1 integrin mAbs. To analyze FBNs and ELN organization into ECM, cells were fixed immunoreacted as described previously [15]. In particular, the FBNs organization was monitored 48 h after seeding: cold methanol fixed cells were reacted for 1 h with 1 μg/ml anti-FBNs mAb, which recognizes all FBN isoforms. The ELN organization was investigated by fixing fibroblasts in 1% PFA for 20 min, treating 1 h at 37°C with 10 U/ml hyaluronidase and immunoreacting for 1 h with 1:50 diluted anti-ELN mAb.

Cells were then incubated for 1 h with anti-mouse or anti-rabbit secondary Abs conjugated to Alexa Fluor 594 and 488, or with anti-goat IgG. IF signals were acquired by a CCD black-and-white TV camera (SensiCam-PCO Computer Optics GmbH, Germany) mounted on a Zeiss fluorescence Axiovert microscope and digitalized by Image Pro Plus software (Media Cybernetics, Silver Spring, MD). All experiments were repeated three times.

### Microarray procedures

Total RNA was extracted from skin fibroblasts of patients and controls using the Qiagen RNeasy kit according to manufacturer’s instructions (Qiagen, Hilden, Germany). RNA quality control was assessed on an Agilent 2100 BioAnalyzer (Agilent Technologies, Santa Clara, CA, USA). Transcriptome-wide expression profiling was performed using the Affymetrix Gene 1.0 ST platform. Microarray analysis was performed starting from 250 ng of total RNA per sample; labeled targets were prepared using Ambion Whole Transcript Expression Kit (Life Technologies) and GeneChip WT Terminal Labeling and Controls Kit (Affymetrix UK Ltd, Wycombe La High Wycombe, UK) in accordance with manufacturers’ instructions. In brief, total RNA was primed with synthetic primers containing a T7 promoter sequence, reverse transcribed into first-strand cDNA and converted into double-stranded cDNA. Following the *in vitro* transcription, cRNA were reverse transcribed and the corresponding cDNA was fragmented, biotin labeled, and hybridized over night at 45° C onto the arrays. The chips were then washed in the Fluidics station FS 450, scanned using the scanner 3000 7G system, and analyzed with the Affymetrix GeneChip Operating Software. Analysis of miRNA expression profile was performed on patients’ and controls’ fibroblasts in accordance with manufacturer’s instructions, starting from 250 ng of total RNA labeled with the Affymetrix Flash Tag Biotin Labeling Kit, followed by the hybridization on the GeneChip miRNA 3.0 array. The resulting CEL files were analyzed using Partek Genomics Suite software, version 6.6 Copyright; 2014 (Partek Inc., St. Louis, MO, USA). One-way ANOVA analysis was conducted to identify the differentially expressed genes (DEGs) between patients and controls by using a combination of fold change value greater than 1.5 and a false discovery rate (FDR) ≤0.3, according to the Benjamini-Hochberg procedure [16]. One-way ANOVA (1.5-fold and uncorrected p≤0.05) was also applied to identify differentially expressed miRNAs between the two groups. To identify significantly perturbed biological processes and enriched pathways in JHS/EDS-HT cells, Partek Pathways algorithm and DAVID functional annotation clustering were queried. In particular, the main Gene Ontology (GO) terms were examined with a p-value ≤0.05 and FDR ≤ 0.3 after Benjamini Hochberg correction.

The miRNA target prediction databases miRWalk, TargetScan, and miRDB were queried to correlate the differentially expressed miRNAs with the DEGs. All microarray data are MIAME compliant, and the raw data have been deposited in the MIAME compliant GEO database with the accession numbers GSE77753 and GSE77756.

### Quantitative real-time PCR

Relative expression levels of a series of selected genes/miRNAs identified by array analysis were confirmed by quantitative real-time PCR (qPCR) by using different RNA extractions. In particular, 3 μg of total RNA were reverse-transcribed with random primers by standard procedure. qPCR were performed with SYBR Green qPCR Master Mix (Life Technologies), 10 ng of cDNA, and with 10 μM of each primers set. qPCR were performed using the ABI PRISM 7500 Real-Time PCR System by standard thermal cycling conditions. *HPRT*, *GAPDH*, *ATP5B*, *CYC1*, and *RPLP0* reference genes were also amplified for normalization of cDNA loading. Relative mRNA expression levels were normalized to the geometric mean of these reference genes and analyzed using the 2^-(ΔΔCt)^ method. Expression of miRNA was assayed using stem-loop RT–PCR starting from 50 ng of total RNA in a final volume of 15 μl followed by TaqMan based qPCR profiling in accordance with manufacturer’s instructions (Life Technologies). The qPCR reaction contained 1.3 μl of reverse transcriptase product, 10 μl of TaqMan 2x Universal PCR Master Mix, and 1 μl of the appropriate TaqMan MicroRNA assay containing primers and probes for the target miRNA. Expression of selected miRNAs was based on the 2^-(ΔΔCt)^ method by using *RNU66* as endogenous control, and qPCR reactions were run in triplicate. Statistical analyses were performed with GraphPad Prism software (GraphPad Software, Inc, La Jolla, CA, USA). Results were expressed as the mean value of relative quantification ± SEM. Statistical significance between groups was determined using one sample t test (*p < 0.05, **p < 0.01, and ***p < 0.001).

## Results

### Clinical findings

Clinical findings of the five women are summarized in Table 1. All patients presented with typical multisystem manifestations also including neurologic, psychiatric, cardiovascular, gastrointestinal, pelvic/gynecologic and immunologic features. All individuals presented widespread chronic musculoskeletal pain of high intensity (i.e., a NRS-11 always above 6/10) and refractory to opioid use.

**Table 1:** Clinical findings of JHS/EDS-HT patients.

	P1	P2	P3	P4	P5
Sex/Age (years)	F/34	F/49	F/36	F/53	F/34
Family history	+	+	+	-	-
Villefranche criteria for EDS-HT	+	+	+	+	-
Brighton criteria	+	+	+	+	+
**MUCOCUTANEOUS**					
Mildly hyperextensible skin	+	+	+	-	+
Velvety/silky/soft skin texture	+	+	+	+	+
*Striae rubrae* and/or *distensae* in young age	+	+	+	-	+
Small or post-surgical atrophic scars	+	+	+	+	+
Light *blue sclerae*	+	+	+	-	+
Easy bruising	+	+	+	+	+
Resistance to local anaesthetics	+	+	-	+	+
**OSTEOARTICULAR**					
Generalized joint hypermobility (BS≥5/9)	+	+	+	+	-
Chronic generalized musculoskeletal pain	+	+	+	+	+
Recurrent dislocations	+	+	+	+	+
Recurrent inflammatory soft-tissue lesions	+	+	+	-	+
Temporomandibular joint dysfunction	+	+	+	+	+
Early osteoarthritis	+	+	+	+	+
**ORTHOPEDIC**					
High arched/narrow palate	+	+	+	+	-
Flat foot	+	+	+	+	+
Mild scoliosis	+	+	+	+	+
Dorsal hyperkyphosis or lumbar hyperlordosis	+	+	+	+	+
*Genua*, *halluces*, or *cubita valga*	+	+	+	+	+
Minor asymmetry at lower limbs and other body areas	+	+	+	-	-
Premenopausal reduced bone mass	+	+	+	+	na
**MUSCULAR**					
Muscle hypotonia	+	+	+	+	+
Recurrent myalgias and cramps	+	+	+	+	+
**GASTROINTESTINAL**					
Gastroesophageal reflux	+	+	+	+	+
Defecatory dysfunction	+	+	-	+	+
Unclassified food intolerances	-	-	-	+	+
Visceroptosis	-	-	-	-	+
**CARDIOVASCULAR**					
Valvular regurgitation with mild hemodynamic involvement	+	+	-	+	-
Mitral valve prolapse	-	+	-	-	-
Raynaud's phenomenon/acrocyanosis/*livedo reticularis*	+	+	na	na	-
**NEUROPSYCHIATRIC**					
Chronic fatigue	+	+	+	+	+
Impaired memory and concentration	+	+	+	+	+
Headache	+	+	+	+	+
Cardiovascular dysautonomia/orthostatic intolerance	+	+	+	+	+
Paresthesias	+	+	+	+	+
Somatosensory amplification	+	+	+	+	+
Obsessive-compulsive trait	na	+	na	+	+
**ALLERGIES**					
Asthma	+	-	-	-	-
Atopic dermatitis	-	na	+	+	-
**UROGYNECOLOGICAL**					
Dysmenorrhea	+	+	-	+	+
Metrorrhagias	+	+	+	+	+
Urinary stress incontinence	+	+	-	+	+
Pelvic prolapse	+	-	+	+	-
**OCULAR**					
Myopia	-	+	+	-	-
Dry eyes	+	+	-	-	+

BS, Beighton score; F, female; na, not available; +, present, -, absent.

### Extracellular matrix organization

**Fig 1:**
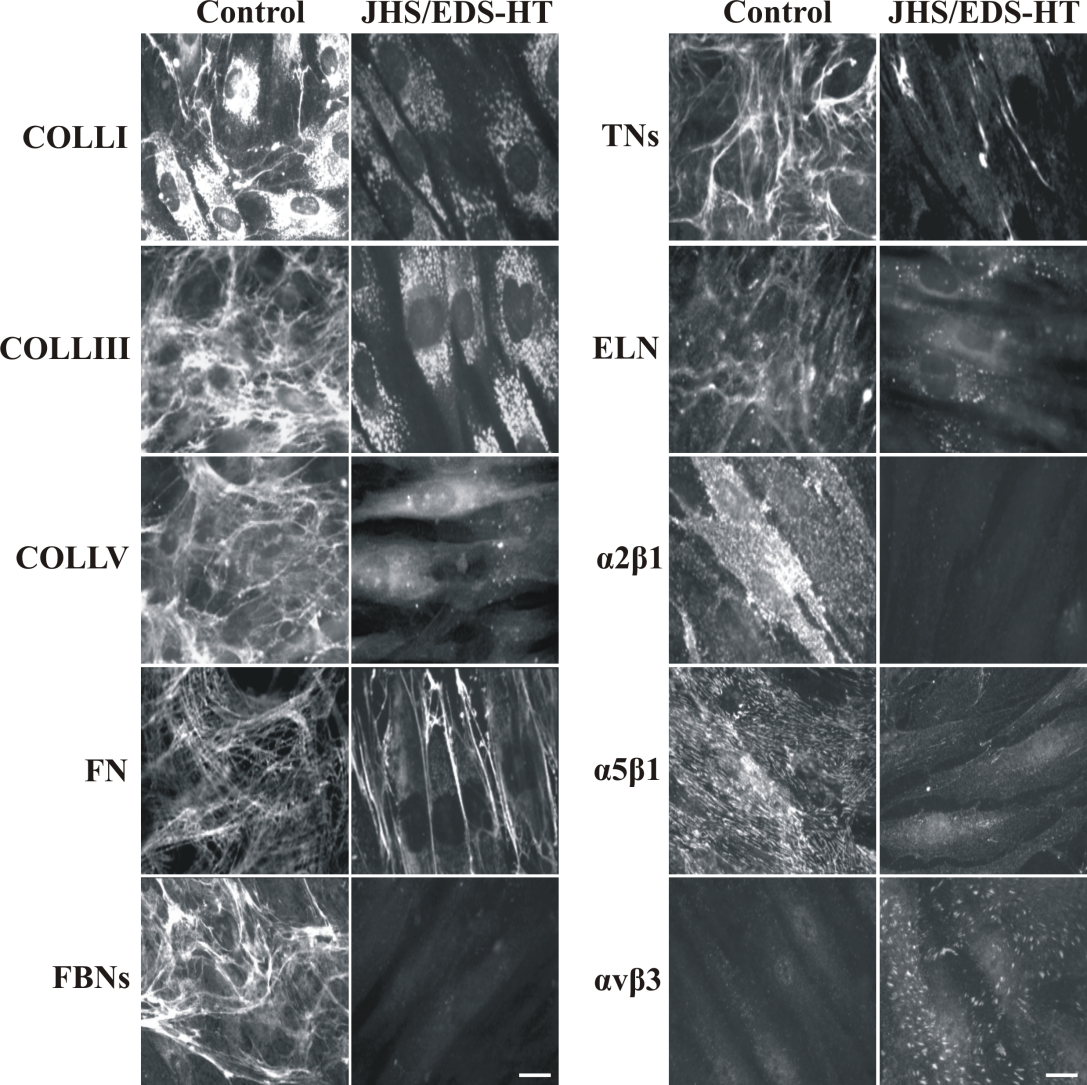
Organization of collagens, other glycoproteins, and their integrin receptors in control and JHS/EDS-HT skin fibroblasts. Left panel: Control and patients’ cells grown for 48 h were analyzed with specific Abs directed against COLLI, COLLIII, and COLLV. FN was investigated by labeling the cells with an Ab recognizing all of FN isoforms. Scale bar: 10 μm. Experiments were repeated three times. The images are representative of three control and five JHS/EDS-HT cell strains. Right panel: TNs were investigated by labeling the cells with a specific Ab recognizing all TN isoforms. The α2β1, α5β1, and αvβ3 integrin receptors were analyzed with specific mAbs recognizing their ligand-binding sites. All of the proteins were investigated 48 h after seeding. IF of ELN was performed on the control and patients’ fibroblasts 7 days after seeding using a specific Ab. Scale bar: 10 μm. Experiments were repeated three times. The images are representative of three control and five JHS/EDS-HT cell strains.

To investigate the organization of different structural proteins into the ECM of JHS/EDS-HT and control skin fibroblasts, expression of COLLI, COLLIII, COLLV, FN, TNs, FBNs and ELN, and distribution of α2β1, α5β1, and αvβ3 integrin receptors were analyzed by IF. As shown in Fig 1, COLLI was accumulated in the cytoplasm with a few thin fibrils into the ECM in control fibroblasts, whereas it was only detected in the cytoplasm at lower levels in patients’ cells. COLLIII and COLLV were assembled into the ECM by control cells, but not by JHS/EDS-HT fibroblasts, in which the proteins were only detected in the cytoplasm. FN, FBNs, and TNs were organized in fibrillar and differently shaped networks covering the control fibroblasts, whereas these proteins were not assembled into the ECM of JHS/EDS-HT cells. In particular, only a few FN and TN fibrils were localized in the intercellular spaces and FBNs were undetectable in patients’ fibroblasts. ELN was organized in a matrix covering 7-day-grown control fibroblasts, whereas it was not assembled into the ECM of JHS/EDS-HT cells, which retained this protein in sparse cytoplasmic spots. JHS/EDS-HT fibroblasts also showed lack of COLL- and FN-specific receptors, α2β1 and α5β1 integrins, respectively, on the cell surface, as compared to control cells. αvβ3 integrin was almost undetectable in controls, whereas, it was organized in linear patches on the cell surface in JHS/EDS-HT cells. IF analyses performed on all patients’ cells, either JHS or EDS-HT, showed the same pattern and comparable disorganization of the ECM proteins and integrin receptors.

### Gene expression profiling

In order to identify genes potentially involved in the molecular mechanisms underlying the complex pathogenetic basis of JHS/EDS-HT, transcriptome-wide expression analysis was carried out comparing gene expression pattern between patients’ and controls’ skin fibroblasts and using the Benjamini-Hochberg procedure to control the false discovery rate [16].

A total of 208 DEGs were identified by applying a fold change threshold ≥1.5 with a FDR ≤0.3. In particular, 46 genes were significantly up-regulated and 162 down-regulated (S1 Table). Panel A in S1 Fig represents the scatter plot of the whole microarray data, and Table 2 shows a selection of DEGs. To group transcripts with similar expression profiles between patients and controls, hierarchical clustering of the DEGs was conducted (Panel B, S1 Fig).

**Table 2:** Selection of DEGs in JHS/EDS-HT skin fibroblasts.

**Down-regulated genes**			
**Gene symbol**	**Gene description**	**p-value**	**Fold change**
***SFRP2***	Secreted frizzled-related protein 2	0,0030	-14,57
***NR4A2***	Nuclear receptor subfamily 4, group A, member 2	0,0002	-8,25
***HES1***	Hes family bHLH transcription factor 1	8.99E-05	-5,48
***PDE1C***	Phosphodiesterase 1C, calmodulin-dependent 70kDa	0,0212	-5,07
***LHX9***	LIM homeobox 9	0,0098	-4,49
***NR4A1***	Nuclear receptor subfamily 4, group A, member 1	0,0005	-4,19
***FLG***	Filaggrin	0,0068	-3,94
***KCNQ5***	Potassium voltage-gated channel, KQT-like subfamily, member 5	0,0012	-3,91
***NPR3***	Natriuretic peptide receptor 3	0,0186	-3,90
***TGM2***	Transglutaminase 2	0,0005	-3,71
***ID1***	Inhibitor of DNA binding 1, dominant negative helix-loop-helix protein	7.7E^-^05	-3,43
***INHBA***	Inhibin, beta A	0,0094	-3,41
***CHRM2***	Cholinergic receptor, muscarinic 2	0,0041	-3,37
***PRICKLE1***	Prickle homolog 1 (Drosophila)	0,0012	-3,26
***FNDC1***	Fibronectin type III domain containing 1	0,0079	-3,23
***GPC4***	Glypican 4	0,0122	-3,17
***MALL***	Mal, T-cell differentiation protein-like	0,0052	-3,15
***SULF1***	Sulfatase 1	0,0016	-3,11
***PRLR***	Prolactin receptor	0,0065	-3,09
***ITGA2***	Integrin, alpha 2	0,0071	-3,05
***CDH2***	Cadherin 2, type 1, N-cadherin (neuronal)	0,0006	-3,05
***SNAI1***	Snail homolog 1 (Drosophila)	0.0015	-2.96
***TRIB1***	Tribbles homolog 1 (Drosophila)	0.0019	-2.92
**Up-regulated genes**			
***IGSF10***	Immunoglobulin superfamily, member 10	0,021	3,13
***CLIC2***	Chloride intracellular channel 2	0,002	3,12
***MIR221***	MicroRNA 221	0,001	2,99
***MIR21***	MicroRNA 21	0,009	2,95
***GSTM5***	Glutathione S-transferase mu 5	0,006	2,85
***AQP9***	Aquaporin 9	2.01E-04	2,82
***AKR1C3***	Aldo-keto reductase family 1, member C3	1.42E-04	2,80
***CLEC2B***	C-type lectin domain family 2, member B	0,011	2,71
***MIR503***	MicroRNA 503	0,017	2,57
***COLEC12***	Collectin sub-family member 12	0,001	2,34
***PARP8***	Poly (ADP-ribose) polymerase family, member 8	0,004	2,21
***CLDN11***	Claudin 11	0,005	2,17
***CFD***	Complement factor D (adipsin)	0,014	2,12
***SELENBP1***	Selenium binding protein 1	4.8E-05	2,03
***ADH1C***	Alcohol dehydrogenase 1C (class I), gamma polypeptide	0.017	2.02
***SLC40A1***	Solute carrier family 40 (iron-regulated transporter), member 1	0.021	1.97
***PPARG***	Peroxisome proliferator-activated receptor gamma	0.011	1.93
***MIR22***	MicroRNA 222	0.001	1.87
***UTS2D***	Urotensin 2 domain containing	0.002	1.86
***LRCH2***	Leucine rich repeats and calponin homology domain containing 2	0.0002	1.85
***AKR1C2***	Aldo-keto reductase family 1, member C2	0.02	1.81

To identify biological processes that are over- or under-represented in patients’ fibroblasts, we classified all up- and down-regulated genes according to the GO categories. This analysis, performed on up-regulated genes, generated 4 distinct GO clusters (Table 3 and S2 Table). The most enriched GO term, i.e., “immune response” and “defense response” processes comprised *APOL1*, *AQP9*, *PTGER4*, *ENPP2*, *PPARG*, *APOBEC3G*, *COLEC12*, and *CFD*. A range of genes, i.e., *AKR1C3*, *AKR1C2*, *PHYHD1*, *MAOB*, *ADH1C*, *ND6*, and *GSTM5* were related to “oxidation reduction process” and involved in different aspects of cellular metabolism, such as “metabolism of xenobiotics by cytochrome P450”. Biological processes related to chemical homeostasis, including *AQP9*, *ADM*, *PPARG*, *CLDN11*, and *SLC40A1* were also over-represented in JHS/EDS-HT fibroblasts.

**Table 3:** Selection of DAVID functional annotation clustering of up-regulated genes.

Cluster	Enrichment Score	Category	Term	p-value
1	3.08	GOTERM_BP_FAT	GO:0045087~innate immune response	1.75E-04
		GOTERM_BP_FAT	GO:0006955~immune response	4.03E-04
		GOTERM_BP_FAT	GO:0006952~defense response	0.007870
2	2.56	KEGG_PATHWAY	hsa00980:Metabolism of xenobiotics by cytochrome P450	2.41E-04
		SP_PIR_KEYWORDS	oxidoreductase	0.004209
		SP_PIR_KEYWORDS	nad	0.005675
		GOTERM_BP_FAT	GO:0055114~oxidation reduction	0.009214
3	0.88	GOTERM_BP_FAT	GO:0048878~chemical homeostasis	0.0201954

Functional analysis of down-regulated genes in JHS/EDS-HT cells yielded 20 different clusters (Table 4 and S3 Table). The significantly under-represented processes included transcripts with transcription regulator activity, i.e., *IL6*, *SMAD7*, *KLF10*, *NR4A2*, *NFKBIA*, *NR4A1*, *NR4A3*, *FOSB*, *JUNB*, *LIF*, *HES1*, *INHBA*, *GATA2*, *FOS*, and involved in regulation of kinase activity, i.e., *SPRY2*, *ERCC6*, *PRLR*, *MET*, *SPHK1*, *TGFA*, *PDGFC*, *DGKI*, *GADD45B*, *TRIB1*, *VLDLR*, and *CBS*. Processes related to regulation of inflammatory and defense responses with different involved transcripts, i.e., *PVR*, *IL6*, *TGM2*, *NFKBIA*, *ITGA2*, and *IDO1*, were also under-represented in patients’ cells. Different down-regulated transcripts, such as *PCDHB8*, *PCDHB16*, *TEK*, *PCDH9*, *ROR2*, *ITGA4*, *CDH2*, *CDH10*, *SHROOM3*, *CHRM2*, *SMAD7*, *FHL2*, *DSP*, *OXTR*, *ITGA2*, *SSX2IP*, and *HOMER1*, were involved in cell adhesion. This GO term was particularly enriched in genes that encode either protocadherins, i.e., *PCDHB8*, *PCDH9*, and *PCDHB16*, or cadherins, i.e., *CDH2*, and *CDH10*, a superfamily of Ca^2+^-dependent transmembrane proteins that act as cell adhesion molecules involved in homophilic cell adhesion and cell-cell junction organization (Table 4 and S3 Table).

**Table 4:** Selection of DAVID functional annotation clustering of down-regulated genes.

Cluster	Enrichment Score	Category	Term	p-value
1	4.29	GOTERM_BP_FAT	GO:0006357~regulation of transcription from RNA polymerase II promoter	2.26E-07
2	3.58	GOTERM_BP_FAT	GO:0043549~regulation of kinase activity	9.52E-04
3	3.06	INTERPRO	IPR001092:Basic helix-loop-helix dimerisation region bHLH	4.96E-05
4	2.98	GOTERM_BP_FAT	GO:0003700~transcription factor activity	8.97E-04
5	2.97	INTERPRO	IPR000837:Fos transforming protein	0.00189
6	2.89	UP_SEQ_FEATURE	domain:Helix-loop-helix motif	3.12E-04
7	2.78	GOTERM_CC_FAT	GO:0005913~cell-cell adherens junction	1.78E-04
		GOTERM_CC_FAT	GO:0005912~adherens junction	2.68E-04
8	2.34	GOTERM_BP_FAT	GO:0051101~regulation of DNA binding	0.001380
		KEGG_PATHWAY	hsa04350:TGF-beta signaling pathway	0.015857
9	2.26	INTERPRO	IPR001781:Zinc finger, LIM-type	0.003321
10	2.04	GOTERM_BP_FAT	GO:0031349~positive regulation of defense response	8.29E-04
11	1.89	INTERPRO	IPR002126:Cadherin	0.013062
		GOTERM_BP_FAT	GO:0007156~homophilic cell adhesion	0.043110
12	1.87	GOTERM_BP_FAT	GO:0042981~regulation of apoptosis	0.002879
13	1.85	GOTERM_BP_FAT	GO:0043408~regulation of MAPKKK cascade	0.004836
		KEGG_PATHWAY	hsa04630:Jak-STAT signaling pathway	0.007322
14	1.78	GOTERM_BP_FAT	GO:0048660~regulation of smooth muscle cell proliferation	0.001149
15	1.77	INTERPRO	IPR003070:Orphan nuclear receptor	2.09E-04

We also analyzed the global expression levels of miRNAs. By selecting a ≥1.5-fold change threshold, 19 miRNAs showed a differential expression relative to controls: 5 were up-regulated and 14 down-regulated (S4 Table). We compared the DEGs list with the differentially expressed miRNAs to investigate a possible correlation between the expressed miRNA and mRNA in JHS/EDS-HT cells (S5 Table). This analysis showed that the up-regulated hsa-miR-378a-3p, hsa-miR-224-5p, hsa-let-7f-5p, and hsa-miR-3609 miRNAs might regulate the transcriptional levels of many down-regulated ECM-related genes and transcription factors, i.e., *SULF1*, *GPC4*, *ITGA4*, *SIK1*, *FOSB*, *NR4A1*, *NR4A2*, and *NR4A3*. The down-regulated hsa-miR-99b-5p, hsa-miR-214-3p, hsa-miR-125a-3p, hsa-miR-664-5p, and hsa-miR-324-5p miRNAs have as potential targets several up-regulated DEGs related to cell adhesion and signal transduction, i.e., *CLDN11*, *AQP9*, *DCLK1*, *FKBP5*, and *FZD3* (S5 Table).

### Pathways enrichment analysis

To identify differentially expressed pathways, enrichment analysis was carried out on all DEGs by using both Partek pathways algorithm and DAVID database with a significance threshold of p-value *<*0.05. This analysis showed perturbation of several signaling transduction pathways that are crucial for correct architecture and homeostasis of various connective tissues (S6 Table). In particular, the PI3K-AKT pathway was under-represented in JHS/EDS-HT cells. This was indicated by the decreased expression of interleukin 6 (*IL6*, -2.03), prolactin receptor (*PRLR*, -3.08), integrin receptors alpha 2 (*ITGA2*, -3.05), and alpha 4 (*ITGA4*, -2.74), G protein-coupled receptor *CHRM2* (-3.36), transcription factor *NR4A1* (-4.19), v-myc avian myelocytomatosis viral oncogene homolog (*MYC*, -1.60), and by different members of receptor tyrosine kinases including MET proto-oncogene-receptor tyrosine kinase (*MET*, -2.11), and TEK tyrosine kinase (*TEK*, -1.63).

Concerning TGFβ signaling, patients’ cells showed decreased expression of genes encoding cytokines, such as inhibin beta A (*INHBA*, -3.40), and intracellular effectors, such as SMAD family member 7 (*SMAD7*, -2.66), and inhibitor of DNA binding 1 (*ID1*, -3.43), and 3 (*ID3*, -2.24).

Pathway analysis also revealed perturbation of JAK-STAT signaling. Different related genes encoding cytokines and receptors, such as *IL6*, leukemia inhibitory factor (*LIF*, -2.26), suppressor of cytokine signaling 3 (*SOCS3*, -1.89), sprouty RTK signaling antagonist 2 (*SPRY2*, -1.63), *PRLR*, and *MYC* mentioned above, were down-regulated.

Pathways analysis also identified perturbation of osteoclast differentiation, as shown by the down-regulation of Fos/activator protein-1 osteoclastogenic transcription factors including Fos (*FOS*, -2.25; and *FOSB*, -2.07) and jun B proto-oncogene (*JUNB*, -1.78). Other dysregulated genes that participate in this pathway were four and a half LIM domains 2 (*FHL2*, -1.59), inhibitor of NFkB signaling (*NFKBIA*, -1.63), and peroxisome proliferator activated receptor γ (*PPARG*, +1.93).

Pathways enrichment analysis also highlighted that the signaling calcium pathways seems to be altered in patients’ fibroblasts. Indeed, different members of this signaling cascade showed a decreased expression including calcium exchanger solute carrier family 8 (Na^+^/Ca^2+^exchanger), member 1 (*SLC8A1*, -2.16), oxytocin receptor (*OXTR*, -2.72), and different second messengers i.e., cholinergic receptor muscarinic 2 (*CHRM2*, -3.36), sphingosine kinase 1 (*SPHK1*, -1.90), and calcium-dependent enzymes such as phosphodiesterase 1C, calmodulin-dependent 70 kDa (*PDE1C*, -5.07), and myosin light chain kinase (*MYLK*, -1.85).

Enrichment analyses further revealed perturbation of pathways involved in regulation of redox homeostasis and xenobiotics metabolism mediated by cytochrome P450 contained only up-regulated genes, such as members of the alcohol dehydrogenase family (*ADH1C*, +2.02), glutathione S-transferase (*GSTM5*, +2.85), flavin monoamine oxidase (*MAOB*, +1.72), and aldo/keto reductase (*AKR1C2*, +1.81; *AKR1C3*, *+*2.80).

### Quantitative real-time PCR validation

**Fig 2:**
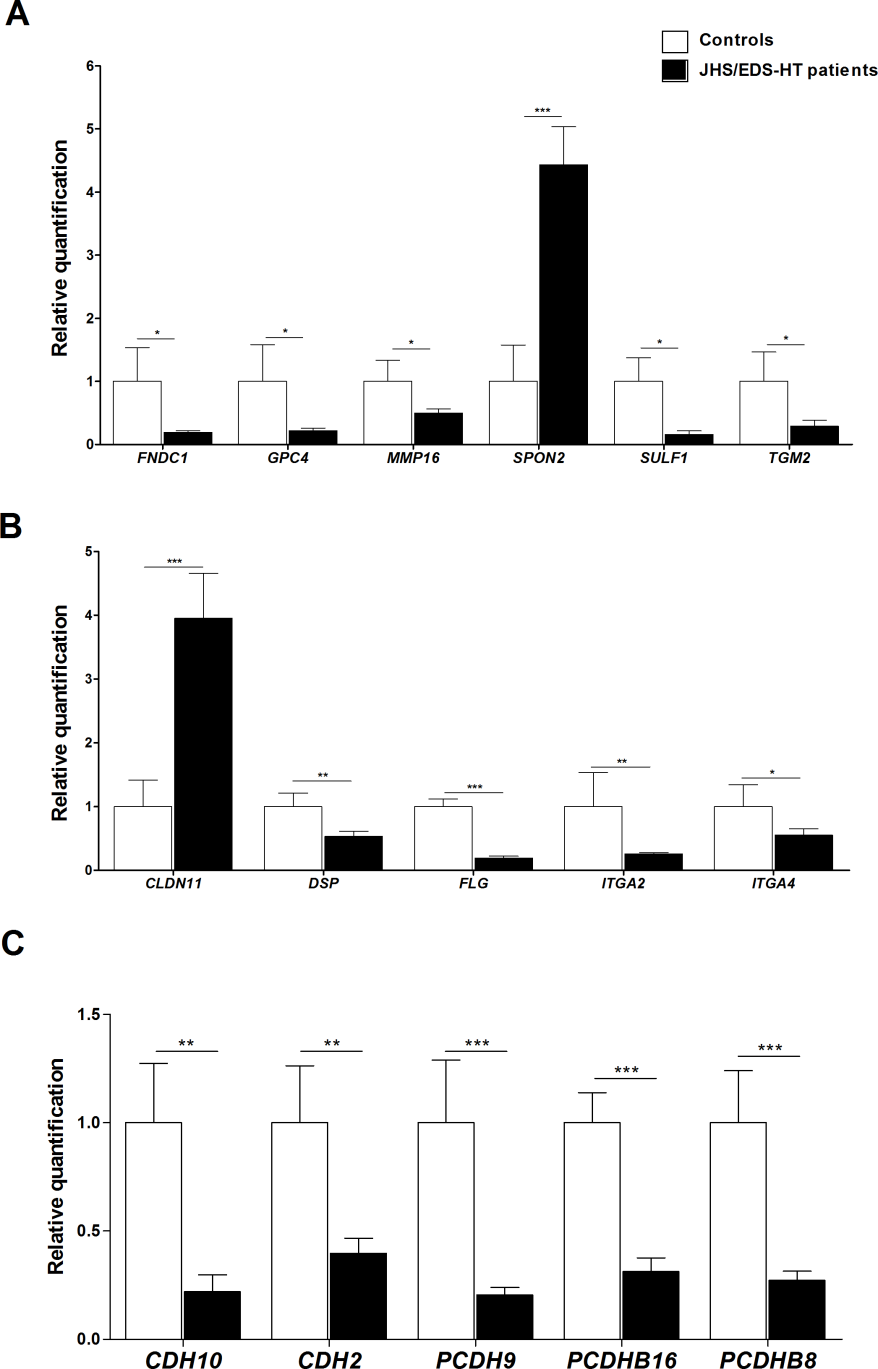
qPCR validation of genes involved in the maintenance of ECM homeostasis. (A) The relative mRNA expression levels of selected genes related to ECM organization, (B) ECM-cell interaction, and (C) genes belonging to cadherins superfamily, were determined with the 2^-(ΔΔCt)^ method normalized with the geometric mean of the *HPRT*, *GAPDH*, *ATP5B*, *CYC1*, and *RPLP0* reference genes. Bars represent the mean ratio of target gene expression in five patients’ fibroblasts compared to five unrelated healthy individuals. qPCR was performed in triplicate, and the results are expressed as mean ± SEM. Statistical significance was calculated with one sample t test (*p<0.05, **p<0.01 and ***p<0.001).

**Fig 3:**
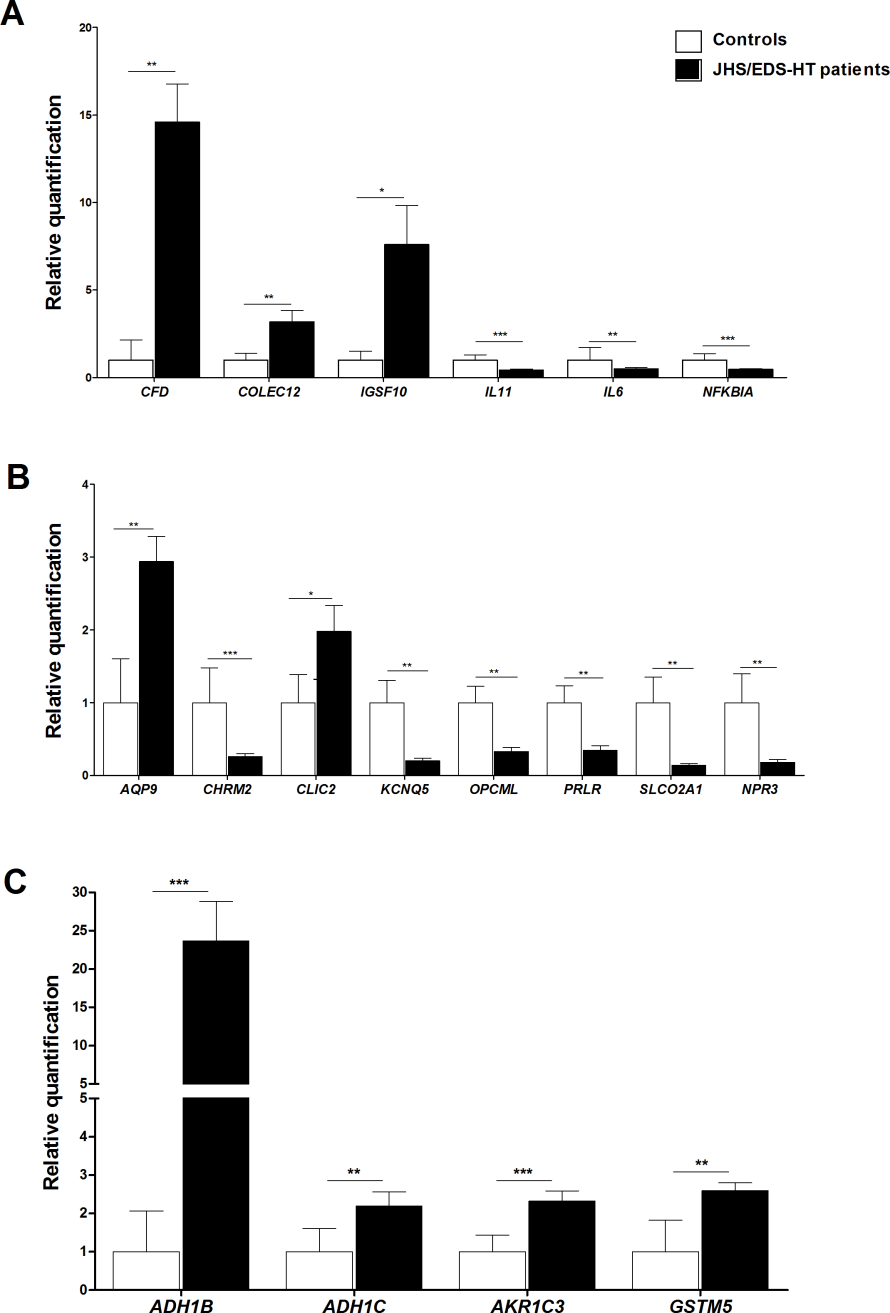
qPCR validation of genes involved in immune and inflammatory responses, signal transduction and energetic/redox homeostasis. (A) The relative mRNA expression levels of selected genes related to immune and inflammation responses, (B) signal transduction, and (C) to energetic/redox homeostasis, were determined with the 2^-(ΔΔCt)^ method normalized with the geometric mean of the five housekeeping genes. Bars represent the mean ratio of target gene expression in five patients’ fibroblasts compared to five unrelated healthy individuals. qPCR was performed in triplicate, and the results are expressed as mean ± SEM. Statistical significance was calculated with one sample t test (*p<0.05, **p<0.01 and ***p<0.001).

**Fig 4:**
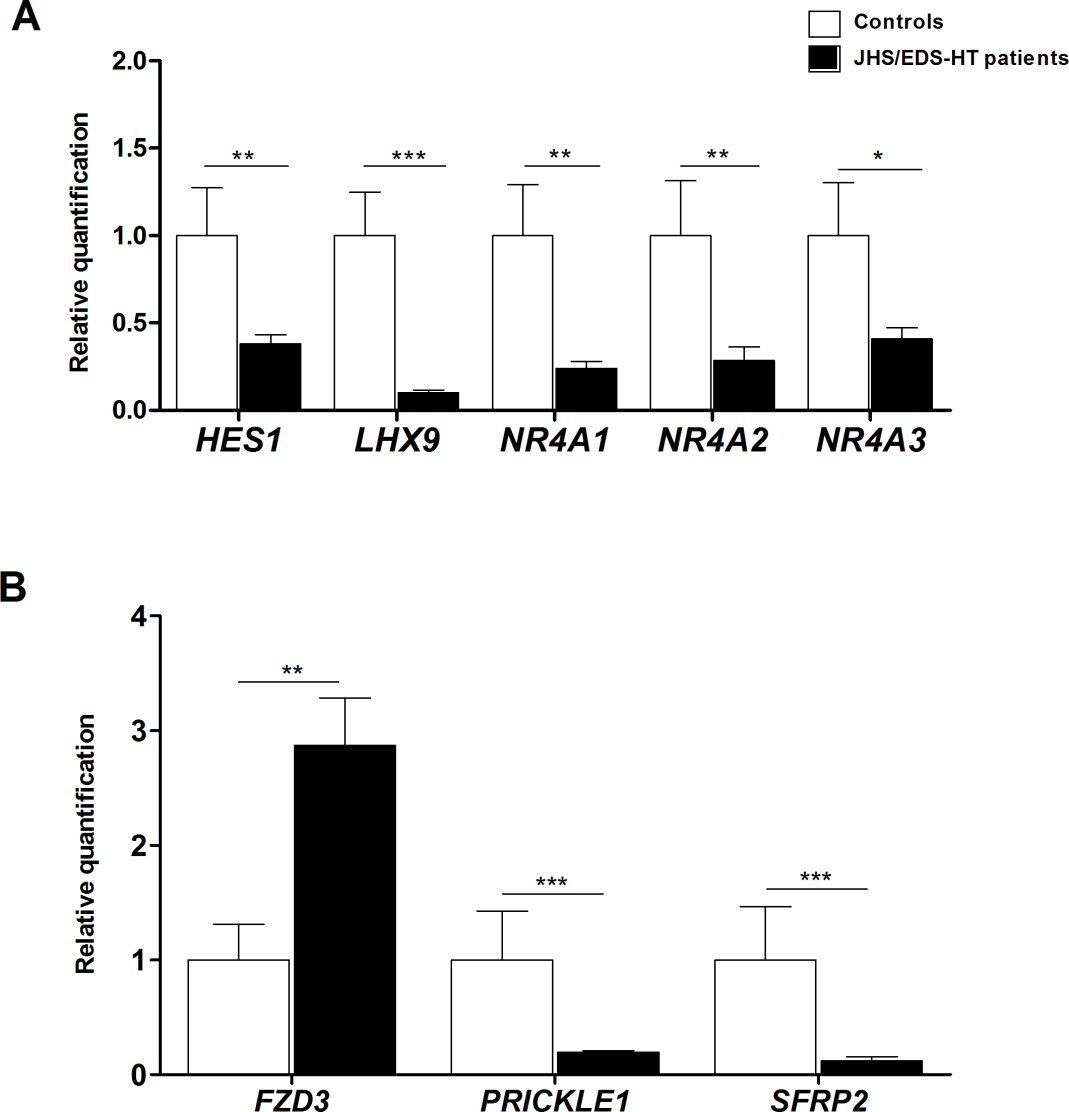
qPCR validation of different transcription factors and genes related to the Wnt signaling. (A) The relative mRNA expression levels of different transcription factors, and (B) genes associated with the Wnt signaling were determined with the 2^-(ΔΔCt)^ method normalized with the geometric mean of the five housekeeping genes. Bars represent the mean ratio of target gene expression in five patients’ fibroblasts compared to five unrelated healthy individuals. qPCR was performed in triplicate, and the results are expressed as mean ± SEM. Statistical significance was calculated with one sample t test (*p<0.05, **p<0.01 and ***p<0.001).

We verified the differential expression of a selection of DEGs/miRNAs by qPCR. Genes were prioritized based on their fold change, GO enrichment analysis, and biological processes significantly perturbed in JHS/EDS-HT cells. We focused on genes involved in maintenance of ECM homeostasis (Fig 2), immune and inflammatory responses, signal transduction and energetic/redox homeostasis (Fig 3), and in regulation of gene transcription and Wnt signaling (Fig 4). qPCR confirmed the marked transcriptional decrease of *FNDC1*, *GPC4*, *MMP16*, *SULF1*, and *TGM2* (Fig 2A). Microarray analysis had indicated a differential expression, although not statistically significant, of *SPON2*, a member of the mindin–F-spondin family of secreted ECM protein. Based on recent evidences that the SPON2 might be a biomarker of osteoarthritis and contribute to activation of innate immunity in allergic airways diseases, we validated its expression by qPCR that demonstrated an approximately 4-fold increase (*p*<0.001). Differential expression of *CLDN11*, *DSP*, *FLG*, *ITGA2*, and *ITGA4* all playing a pivotal role in ECM-cell interaction and cell adhesion, were also confirmed (Fig 2B). qPCR showed decreased expression of transcripts encoding members of the cadherin superfamily, including *CDH10*, *CDH2*, *PCDH9*, *PCDHB16*, and *PCDHB8* (Fig 2C). The expression changes of a range of transcripts implicated in immune and inflammatory responses, including *CFD*, *COLEC12*, *IGSF10*, *IL11*, *IL6*, and *NFKBIA* were confirmed (Fig 3A). We also confirmed significant variations in the expression pattern of transcripts involved in signal transduction and with functions related to transport activity that are *AQP9*, *CHRM2*, *CLIC2*, *KCNQ5*, *OPCML*, *PRLR*, *SLCO2A1*, and *NPR3* (Fig 3B). All were down-regulated except for *AQP9* and *CLIC2* that were up-regulated. qPCR also showed up-regulation of transcripts involved in cellular metabolism and detoxification processes, including *ADH1B*, *ADH1C*, *AKR1C3*, and *GSTM5* (Fig 3C), down-regulation of *HES1*, *LHX9*, *NR4A1*, *NR4A2*, and *NR4A3* transcription factors (Fig 4A), and perturbation of Wnt signaling pathway, as shown by the expression changes of *FZD3*, *PRICKLE1*, and *SFRP2* (Fig 4B).

Finally, qPCR confirmed the differential expression of the most up- and down-regulated miRNAs, comprising hsa-miR-378a, hsa-miR-224, hsa-miR-let-7f, hsa-miR-23a, hsa-miR-27a, and hsa-miR-21 (S2 Fig). The modulation of expression of several miRNAs and the compelling correlation between miRNA-DEGs levels (S5 Table) suggests that epigenetic mechanisms may be involved in the altered gene expression observed in JHS/EDS-HT cells, which merits future studies.

## Discussion

This is the first work that reports on gene expression abnormalities in JHS/HT-EDS skin fibroblasts. Although the sample size is small and findings will therefore need to be confirmed in other patients, our results provide a step forward towards understanding of the complex pathogenetic basis of this condition. Clinical presentation of the present patients reflects the multisystem phenotype of many adults with JHS/EDS-HT [7, 17, 18], since they share a myriad of features affecting connective tissue that range from skin hyperextensibility, gJHM, muscle hypotonia, recurrent myalgia and cramps, chronic pain, pelvic prolapse, gastrointestinal dysfunction, together with still poorly defined inflammatory soft-tissue lesions and atopic signs.

Protein studies revealed a widespread disarray of different ECM structural proteins and marked disorganization of COLLs and FN ECMs, and their specific integrin receptors in patients’ fibroblasts. These findings are consistent with our previous results obtained in classic and vascular EDS patients’ fibroblasts. In particular, the abundant expression of αvβ3 in JHS/EDS-HT fibroblasts further supports the cell survival role of this integrin, which rescues cells from *anoikis* induced by ECM disassembly due to *COL5A1* and *COL3A1* mutations, respectively [13, 19]. Analogous ECM anomalies were reported in other rare EDS types [14, 20–23], suggesting that defective ECM organization is a common feature of EDS skin fibroblasts, irrespective of the underlining molecular defects. Altered ECM assembly shown in this *in vitro* model should reproduce both dermal ultrastructural anomalies, such as irregular and loosely packed collagen fibrils identified in diverse EDSs [24, 25], and structural impairment of different affected connective tissues in JHS/EDS-HT patients including joints, ligaments, tendon, skin, mucosae, muscle, and bone [17, 26–29].

In the attempt to identify significant gene expression changes and molecular processes that may be involved in the pathomechanisms of JHS/EDS-HT, a transcriptome-wide expression profiling was performed. Describing a global mRNA status in a single article is an impossible goal, thus we report only on a selection of DEGs/molecular processes implicated in ECM architecture, skin barrier function, inflammatory/immune and pain responses, and maintenance of homeostasis, correlating well with the systemic manifestations of JHS/EDS-HT patients.

### ECM organization, cell adhesion, and connective tissue integrity

Patients’ cells showed a decreased expression of *TGM2* that encodes transglutaminase 2, a multifunctional enzyme that plays a key role in ECM remodeling, cell adhesion, stabilization of dermal microfibrils, and formation of the basement membrane laminin–nidogen complex [30]. On the other hand, we found increased expression of spondin 2 (*SPON2*) that encodes an ECM protein that functions as a regulator of cellular growth, differentiation, and apoptosis, and plays a critical role in innate immune response by acting as an opsonin to direct macrophage phagocytosis [31, 32]. Accumulated evidences support the role of SPON2 in production of pro-inflammatory cytokines, and development of airway hyper-responsiveness, which may contribute to the severity of allergic airways disease including asthma [33–36]. SPON2 is considered a biomarker of osteoarthritis, since its expression was increased in synovial fluid of patients [37]. In this regard, while in the general population the link between congenital joint laxity and premature joint damage is unclear, this association seems likely in JHS/EDS-HT, as recurrent joint macro- and microtraumatisms are more common in these patients [4]. Moreover, the ensuing early and polyarticular chondral damage is probably one of the very first steps acting in the evolution of musculoskeletal pain [18].

The disarray of different ECM structural proteins and the altered expression of genes involved in ECM remodeling should play a role not only in the generation of inflammation, but also in the neuronal plasticity and hyperexcitability of nociceptive neurons and in the chronification of pain observed in JHS/EDS-HT patients [38]. It is well established that ECM molecules or fragments released by matrix metallopeptidases activating in response to injury and integrin-mediated responses, could modulate inflammatory pathways and hyperalgesic signaling [39, 40].

Cell adhesion, a fundamental process for formation and maintenance of tissues morphogenesis, seems to be altered in JHS/EDS-HT fibroblasts. In particular, we observed an increased expression of *CLDN11*, which encodes a member of the claudin family that are molecules related to tight junctions, which are fundamental for maintenance of tissues architecture and morphogenesis [41]. On the other side, *DSP* that encodes desmoplakin, a member of cytolinker proteins family, showed decreased expression in patients’ cells. Desmoplakin is essential in epidermal sheet formation and is required for assembly of functional desmosomes, maintaining cytoskeletal architecture and reinforcing membrane attachments allowing for stable intercellular adhesion [42]. As a further clue for cell adhesion perturbation, we observed down-regulation of many genes belonging to the cadherin superfamily including *CDH2*, *CHD10*, *PCDH9*, *PCDHB16*, and *PCDHB18*. During embryonic development, cadherins control separation of distinct tissue layers, formation of tissue boundaries, and synapses between neurons. In adult tissues, they are involved in orderly turnover of rapidly growing tissues, such as lining of the gut and the epidermis, regulation of epithelial and endothelial cell junctions, and maintenance of the stable tissue organization [43]. Cadherins also affect numerous signaling pathways, including Wnt-, receptor tyrosine kinases-, NFκB-, and JAK-STAT-signaling [44]. Notably, our findings indicated that dysregulation of different cadherins-related pathways, including JAK-STAT, PI3K-AKT and Wnt signaling cascades, might occur in JHS/EDS-HT cells. This suggests that defective cadherins function may reflect on signaling cascades required for homeostatic regulation of cell survival, differentiation, and proliferation during tissue development and organogenesis.

### Skin barrier function

Microarray revealed differential expression of several genes involved in epidermal development and keratinocyte differentiation, such as *FLG*, *AQP9*, and *AKR1C3*. JHS/EDS-HT cells showed decreased expression of *FLG* that encodes filaggrin, a key protein that plays a central role in the formation of cornified cell envelope, which is critical for an effective skin barrier [45]. Filaggrin aggregates the keratin cytoskeleton to facilitate flattening of keratinocytes in the outermost skin layer [46]. Mutations in *FLG* confer risk for associated allergic diseases including food allergy, and atopic asthma and are the most significant known genetic risk factor for the atopic dermatitis (AD) development, a common chronic inflammatory skin disease characterized by epidermal barrier dysfunction and immunological alterations [47, 48]. Multiple transcriptome data indicated among the AD-related genes the marked down-regulation of *FLG*, suggesting that dysfunction of pathways involved in skin barrier integrity, including keratinocyte differentiation may contribute to the AD pathogenesis [49]. Consistent with these findings, decreased expression of *FLG*, together with other unrecognized genetic and environmental factors, may contribute to a defective epidermal barrier increasing risk of atopic asthma and AD in JHS/EDS-HT patients. In this regard, earlier studies suggested increased prevalence of atopy and asthmatic symptoms in patients with different EDS types also comprising JHS/EDS-HT, in association with various pulmonary physiological abnormalities [50]. On the other hand, patients’ cells showed increased expression of *AKR1C3* and *AQP9*: *AKR1C3* encodes an enzyme of aldo-keto reductase family that promotes inflammation in skin lesions of AD patients [51], while *AQP9* codes for a member of the aquaporins that enhances skin barrier function and antimicrobial defenses [52]. Furthermore, a microarray study on peripheral blood mononuclear cells of patients with irritable bowel syndrome, psoriasis, and rheumatoid arthritis, identified *AQP9* as a novel marker of chronic inflammation underlying these diseases [53].

### Inflammatory, immune and pain responses

Transcriptome data also highlighted significant expression changes of several genes related to inflammatory and immune responses that include *CFD*, *COLEC12*, *NR4A1*, *NR4A2*, *NR4A3*, and *HES1*. JHS/EDS-HT cells displayed an increased expression of complement factor D (*CFD*), a component of the alternative complement pathway that has a role in the inflammatory response and tissue injury [54]. This serine protease was recently found up-regulated in muscle biopsies of patients with Ullrich congenital muscular dystrophy, as a consequence of inflammatory processes [55]. In agreement with our expression data, a recent proteomic analysis showed in JHS/EDS-HT patients’ sera increased levels of different proteins of the complement system, including C9, C1R, and vitronectin, thereby suggesting the possibility of a locally occurring inflammatory process in JHS/EDS-HT patients [56]. CFD and other adipokines are also involved in pathophysiological mechanisms related to osteoarthritis progression and outcome. Regarding this aspect, Martel-Pelletier et al. [57] demonstrated in patients with osteoarthritis a meaningful correlation between high serum levels of CFD and leptin with more cartilage damage, knee osteoarthritis progression and higher incidence of total knee replacement. *COLEC12* codes for a cell surface glycoprotein that acts as a scavenger receptor that is involved in the clearance of glycoproteins released by degranulation of neutrophils at sites of inflammation [58]. *NR4A1*, *NR4A2*, and *NR4A3* are ligand-dependent transcription factors that modulate NF-kB activity in a dynamic fashion, either repressing or enhancing target gene expression leading to altered inflammatory outcome [59]. *HES1* is an early marker of differentiation of multiple endocrine cell types in the developing stomach and gut, which transcriptional activity is regulated by SFRP2, a member of the secreted frizzled-related proteins that can modulate Wnt signaling [60]. In JHS/EDS-HT patients’ cells some negative regulators of the Wnt pathway including *SFRP2*, and *PRICKLE1* were down-regulated, whereas receptor frizzled 3 (*FZD3*), required for the Wnt signaling cascade, showed an increased expression. These findings suggest that a perturbation of this transduction pathway, which is known to be involved in the development and renewal of the intestinal epithelium [61], might also participate in the pathomechanisms underlying the different gastrointestinal dysfunctions, i.e., gastroesophageal reflux, recurrent abdominal pain, defecatory dysfunction, and unclassified food intolerances of JHS/EDS-HT patients [18].

Concerning pain, *PRLR* encodes prolactin receptor, a member of the cytokine receptor superfamily that is expressed in a variety of immune cells, in which this hormone can be pro-inflammatory or anti-inflammatory by regulating proliferation, survival, and release of inflammatory mediators [62]. Serum elevated prolactin (PRL) levels were associated with a variety of pain conditions as migraine, burning, rheumatoid arthritis, and osteoarthritis [63]. PRL can also be released by stimulated sensory neurons and can modulate the activity of nociceptors, thus playing an important role in pain responses and inflammation [63]. Thus, it is reasonable to suppose that the PRL/PRLR system might be involved in the complex mechanisms implicated in the nociceptive and neuropathic pain that, in turn, likely contributes to the widespread chronic pain observed in JHS/EDS-HT patients [4]. About this, in the last years different clinical research attempted to explain the type of chronic pain in JHS/EDS-HT patients and, in particular, the presence of neuropathic pain. Rombaut et al. [64] showed that approximately half of the JHS/EDS-HT patients most likely suffer from neuropathic pain, in accordance with Camerota et al. [65]. In addition, cutaneous innervation involvement associated with a small fiber neuropathy has been recently demonstrated in JHS/EDS-HT patients [66, 67], in line with earlier studies that showed higher prevalence of neuropathic symptoms, such as paresthesias/numbness in hands and/or feet [68, 69]. *KCNQ5* is a member of the K^+^ channels family involved in attenuation of the thermal hyperalgesia-induced inflammatory pain [70]. *ADM* codes for adrenomedullin that is an important mediator for pathological pain, as its expression is enhanced both in acute and chronic inflammation, which triggers up-regulation of pronociceptive mediators and down-regulation of pain-inhibiting molecule in a cascade contributing to the development of morphine tolerance [71]. Of note, all analyzed JHS/EDS-HT patients suffer from chronic generalized musculoskeletal pain and are refractory to opioid use.

### Maintenance of homeostasis

Our results showed up-regulation of several metabolic genes related to oxidant/antioxidant balance, i.e., *GSTM5*, *ADH1B*, *ADH1C*, *SELENBP1*, and *MAOB*. *GSTM5*, encoding glutathione S-transferase mu 5, plays a role in detoxification of drugs and products of oxidative stress [46]. Likewise, *ADH1B*, and *ADH1C*, encode enzymes of alcohol dehydrogenase family that metabolize a wide variety of substrates, including lipid peroxidation products [72]. Selenium-binding protein-1 (*SELENBP1*) is involved in selenium transport, an essential nutrient which displays neuroprotective and antioxidant activities in preventing certain neurologic diseases, such as schizophrenia and bipolar disorder. In this regard, up-regulated expression of *SELENBP1* has been reported in both blood and brain of schizophrenic patients resulting a strong candidate biomarker for schizophrenia [73]. Of note, many JHS/EDS-HT patients show several neuropsychiatric manifestations including mood disorder, reactive depression, maniac depressive illness, anxiety and, perhaps, obsessive-compulsive traits [74, 75]. *MAOB* encodes a monoamine oxidase responsible for the oxidative deamination of different neurotransmitters, such as serotonin, melatonin and dopamine. Increased expression of this enzyme was reported in age-related neurodegenerative diseases wherein it is associated with oxidative stress and vulnerability of the brain dopamine system [76].

In conclusion, although our data were obtained in a connective tissue cell model, this study pointed out significant gene expression changes that should perturb numerous biological processes, finally leading to the systemic clinical manifestations of JHS/EDS-HT patients. Future investigations on a larger cohort of patients are needed to corroborate the present results and also to identify potential biomarkers that may be supportive to the clinical diagnosis of this neglected disorder.

## Supporting Information

S1 Fig Volcano plot and hierarchical clustering. (A) Volcano plot depicts all statistically significant DEGs identified in JHS/EDS-HT cells. The fold-change of DEGs on the x-axis *vs* the statistical significance (*p*-value <0.05, FDR ≤0.3) on the y-axis is shown; up-regulated genes are reported in red, and down-regulated genes are in blue. (B) Hierarchical clustering of 208 DEGs identified in patients’ skin fibroblasts. Red color represents high gene expression, and blue indicates low gene expression. P: patients; C: controls.
(TIF)

S2 Fig Validation of differential expressed miRNAs by qPCR. The expression levels of a selection of up-regulated and down-regulated miRNAs were evaluated using *RNU66* as an internal normalization transcript. The bars represent the mean ratio of the target miRNA expression in patients’ fibroblasts compared with five unrelated healthy individuals. qPCR was performed in triplicate, and the results are expressed as the mean ± SEM. Statistical significance was calculated with one sample t test (*p<0.05, **p<0.01 and ***p<0.001).
(TIF)

S1 Table List of differentially expressed genes in JHS/EDS-HT skin fibroblasts. (XLSX)

S2 Table DAVID clusters of up-regulated genes in JHS/EDS-HT fibroblasts. (XLS)

S3 Table DAVID clusters of down-regulated genes in JHS/EDS-HT fibroblasts. (XLS)

S4 Table List of differentially expressed miRNAs in JHS/EDS-HT skin fibroblasts. (XLS)

S5 Table miRNA-mRNA interaction. (DOC)

S6 Table Top canonical pathways perturbed in JHS/EDS-HT skin fibroblasts. (DOCX)

## Abbreviations

AD: Atopic Dermatitis
BS: Beighton score
COLLs: collagens
COLLI: collagen type I
COLLIII: collagen type III
COLLV: collagen type V
DEGs: differentially expressed genes
EDS: Ehlers-Danlos syndrome
EDS-HT: Ehlers-Danlos syndrome hypermobility type
ELN: elastin
ECM: extracellular matrix
FDR: false discovery rate
FBNs: fibrillins
FN: fibronectin
GO: Gene Ontology
gJHM: generalized joint hypermobility
HCTD: heritable connective tissue disorder
IF: immunofluorescence microscopy
JHS/EDS-HT: Joint Hypermobility syndrome/Ehlers-Danlos syndrome hypermobility type
miRNA: microRNA
Abs: monoclonal antibodies
NRS: numerical rating scale
Ab: polyclonal antibody
qPCR: quantitative real-time polymerase chain reaction
TNs: tenascins
