# Supplementary figures and images for: Transcriptome-Wide Expression Profiling in Skin Fibroblasts of Patients with Joint Hypermobility Syndrome/Ehlers-Danlos Syndrome Hypermobility Type
Source: PLoS One. 2016 Aug 12;11(8):e0161347. doi: 10.1371/journal.pone.0161347 (PMC4982685; doi:10.1371/journal.pone.0161347)

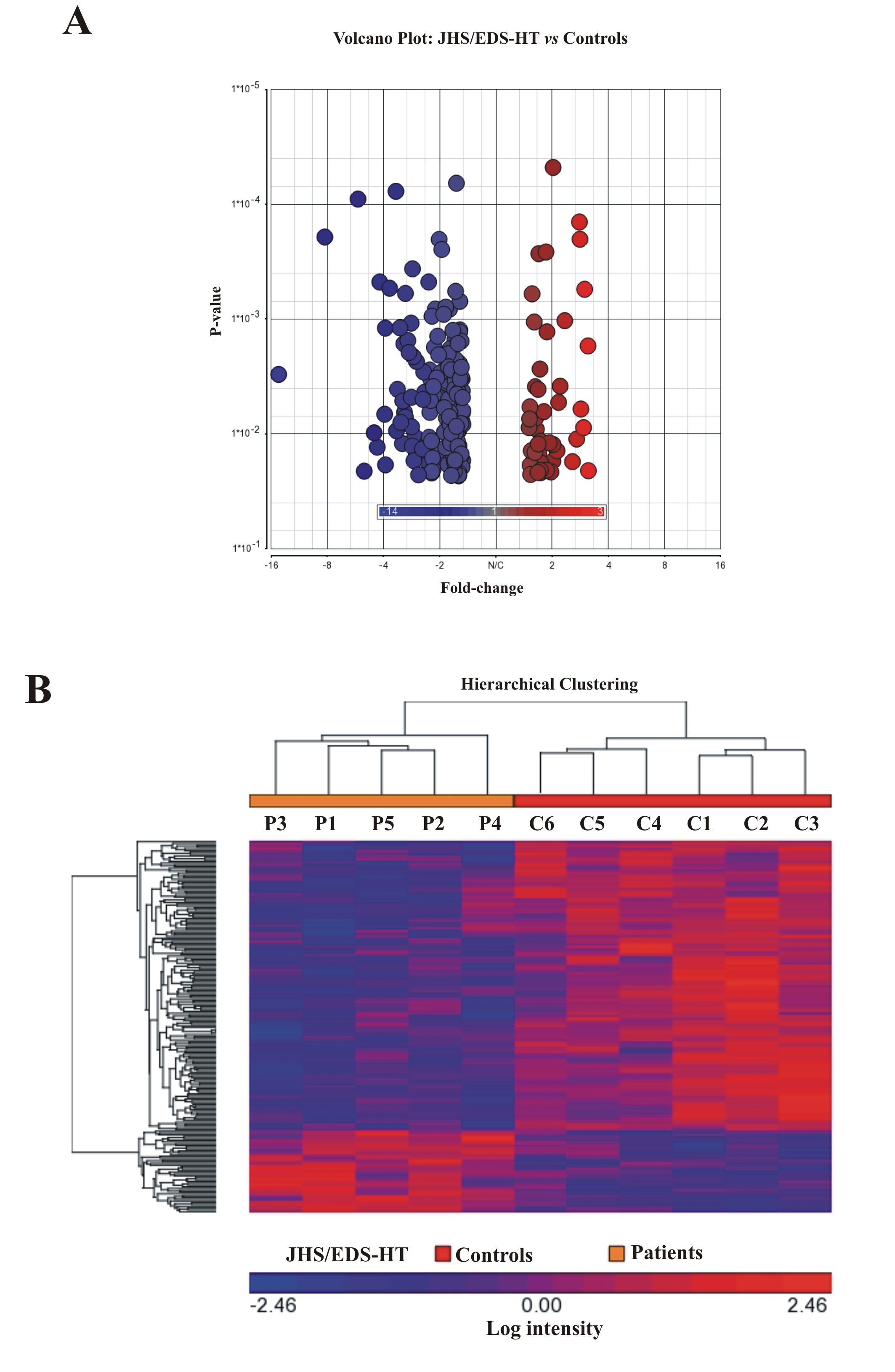

Supplement: S1 Fig — (A) Volcano plot depicts all statistically significant DEGs identified in JHS/EDS-HT cells. The fold-change of DEGs on the x-axis vs the statistical significance (p-value <0.05, FDR ≤0.3) on the y-axis is shown; up-regulated genes are reported in red, and down-regulated genes are in blue. (B) Hierarchical clustering of 208 DEGs identified in patients’ skin fibroblasts. Red color represents high gene expression, and blue indicates low gene expression. P: patients; C: controls. (TIF) [file pone.0161347.s001.TIF]
