# Supplementary material for: Transcriptome-Wide Expression Profiling in Skin Fibroblasts of Patients with Joint Hypermobility Syndrome/Ehlers-Danlos Syndrome Hypermobility Type
Source: PLoS One. 2016 Aug 12;11(8):e0161347. doi: 10.1371/journal.pone.0161347 (PMC4982685; doi:10.1371/journal.pone.0161347)
Supplement: S5 Table — (DOC) [file pone.0161347.s007.doc]

| **S5 Table: miRNA-mRNA interaction**   |  | | --- | |  | | | | | | | | |
| --- | --- | --- | --- | --- | --- | --- | --- | --- | --- |
| **miRNA** | **Fold-change** | **Target symbol^a^** | **Gene description** | **Fold-change** | **Target symbol^a^** | **Gene description** | **Fold-change** |
| **hsa-miR-378-3p** | 3.85 | ***FKBP5*** | FK506 binding protein 5 | 1.74 | ***DACT1*** | Dapper, antagonist of beta-catenin, homolog 1 | -2.84 |
|  |  |  |  |  | ***SULF1*** | Sulfatase 1 | -3.11 |
|  |  |  |  |  | ***ZFPM2*** | Zinc finger protein, multitype 2 | -2.04 |
| **hsa-miR-224-5p** | 3.29 |  |  |  | ***ENC1*** | Ectodermal-neural cortex 1 (with BTB-like domain) | -1.60 |
|  |  |  |  |  | ***FOSB*** | FBJ murine osteosarcoma viral oncogene homolog B | -2.07 |
|  |  |  |  |  | ***GPC4*** | Glypican 4 | -3.17 |
|  |  |  |  |  | ***ID3*** | Inhibitor of DNA binding 3, dominant negative helix-loop-helix protein | -2.24 |
|  |  |  |  |  | ***NR4A1*** | Nuclear receptor subfamily 4, group A, member 1 | -4.19 |
|  |  |  |  |  | ***NR4A3*** | Nuclear receptor subfamily 4, group A, member 3 | -2.24 |
|  |  |  |  |  | ***OPCML*** | Opioid binding protein/cell adhesion molecule-like | -2.29 |
|  |  |  |  |  | ***SERPINE1*** | Serpin peptidase inhibitor, clade E (nexin, plasminogen activator inhibitor type 1), member 1 | -1.89 |
|  |  |  |  |  | ***SFRP2*** | Secreted frizzled-related protein 2 | -14.57 |
|  |  |  |  |  | ***MAFF*** | V-maf musculoaponeurotic fibrosarcoma oncogene homolog F | -1.60 |
|  |  |  |  |  | ***KLF10*** | Kruppel-like factor 10 | -1.97 |
|  |  |  |  |  | ***TRIB1*** | Tribbles homolog 1 | -2.92 |
| **hsa-let-7f-5p** | 2.44 | ***FZD3*** | Frizzled homolog 3 | 1.62 | ***AFF2*** | AF4/FMR2 family, member 2 | -1.76 |
|  |  |  |  |  | ***ARG2*** | Arginase, type II | -1.70 |
|  |  |  |  |  | ***ERCC6*** | Excision repair cross-complementation group 6 | -1.65 |
|  |  |  |  |  | ***RGS16*** | Regulator of G-protein signaling 16 | -1.57 |
| **hsa-miR-3609** | 1.57 | ***FZD3*** | Frizzled homolog 3 | 1.62 | ***CHMP1B*** | Chromatin modifying protein 1B | -2.03 |
|  |  | ***PARP8*** | Poly (ADP-ribose) polymerase family, member 8 | 2.21 | ***CHRM2*** | Cholinergic receptor, muscarinic 2 | -3.37 |
|  |  | ***ST8SIA2*** | ST8 alpha-N-acetyl-neuraminide alpha-2,8-sialyltransferase 2 | 1.74 | ***EBF3*** | Early B-cell factor 3 | -1.64 |
|  |  |  |  |  | ***GPC4*** | Glypican 4 | -3.17 |
|  |  |  |  |  | ***GPR137C*** | G protein-coupled receptor 137C | -1.58 |
|  |  |  |  |  | ***IGF2BP*** | Insulin-like growth factor 2 mRNA binding protein 1 | -1.71 |
|  |  |  |  |  | ***ITGA4*** | Integrin, alpha 4 | -2.73 |
|  |  |  |  |  | ***LIF*** | leukemia inhibitory factor | -2.26 |
|  |  |  |  |  | ***SERPINE1*** | Serpin peptidase inhibitor, clade E (nexin, plasminogen activator inhibitor type 1), member 1 | -1.89 |
|  |  |  |  |  | ***SIK1*** | Salt-inducible kinase 1 | -1.89 |
|  |  |  |  |  | ***SLC1A4*** | Solute carrier family 1 (glutamate/neutral amino acid transporter), member 4 | -1.82 |
|  |  |  |  |  | ***ZFPM2*** | Zinc finger protein, multitype 2 | -2.04 |
|  |  |  |  |  | ***RAB3B*** | RAB3B, member RAS oncogene family | -1.81 |
|  |  |  |  |  | ***OSBPL8*** | Oxysterol binding protein-like 8 | -1.54 |
|  |  |  |  |  | ***PDE5A*** | Phosphodiesterase 5°, cGMP-specific | -2.58 |
|  |  |  |  |  | ***PDGFC*** | Platelet derived growth factor C | -1.50 |
|  |  |  |  |  | ***STK32B*** | Serine/threonine kinase 32B | -2.83 |
| **hsa-miR-99b-5p** | -1.63 | ***CLDN11*** | Claudin 11 | 2.17 | ***TRIB1*** | Tribbles homolog 1 | -2.92 |
| **hsa-miR-3944-5p** | -1.72 |  |  |  | ***ENC1*** | Ectodermal-neural cortex 1 (with BTB-like domain) | -1.59 |
| **hsa-miR-214-3p** | -1.74 | ***ARHGAP42*** | Rho GTPase activating protein 42 | 1.74 | ***CHPF*** | Chondroitin polymerizing factor | -1.67 |
|  |  | ***DCLK1*** | Doublecortin-like kinase 1 | 1.52 | ***FOSB*** | FBJ murine osteosarcoma viral oncogene homolog B | -2.07 |
|  |  | ***FKBP5*** | FK506 binding protein 5 | 1.74 |  |  |  |
|  |  |  |  |  | ***SLC8A1*** | Solute carrier family 8 (sodium/calcium exchanger), member 1 | -2.16 |
|  |  |  |  |  | ***SULF1*** | Sulfatase 1 | -3.11 |
|  |  |  |  |  | ***TSPAN11*** | Tetraspanin 11 | -1.66 |
|  |  |  |  |  | ***SSX2IP*** | Synovial sarcoma, X breakpoint 2 interacting protein | -1.79 |
|  |  |  |  |  | ***SLC1A4*** | Solute carrier family 1 (glutamate/neutral amino acid transporter), member 4 | -1.82 |
|  |  |  |  |  | ***BHLHE40*** | Basic helix-loop-helix family, member e40 | -2.41 |
|  |  |  |  |  | ***PDE5A*** | Phosphodiesterase 5°, cGMP-specific | -2.58 |
|  |  |  |  |  | ***STK32B*** | Serine/threonine kinase 32B | -2.83 |
| **hsa-miR-193-5p** | -1.75 |  |  |  | ***SULF1*** | Sulfatase 1 | -3.11 |
| **hsa-miR-432-5p** | -1.92 |  |  |  | ***OPCML*** | Opioid binding protein/cell adhesion molecule-like | -2.29 |
|  |  |  |  |  | ***SLC8A1*** | Solute carrier family 8 (sodium/calcium exchanger), member 1 | -2.16 |
|  |  |  |  |  | ***PLK3*** | Polo-like kinase 3 | -1.64 |
|  |  |  |  |  | ***SERPINE1*** | Serpin peptidase inhibitor, clade E (nexin, plasminogen activator inhibitor type 1), member 1 | -1.89 |
| **hsa-miR-324-5p** | -2.29 | ***AQP9*** | Aquaporin 9 | 2.82 | ***RNF185*** | Ring finger protein 185 | -1.73 |
|  |  |  |  |  | ***SHROOM3*** | Shroom family member 3 | -2.18 |
| **hsa-miR-664-5p** | -2.47 | ***DCLK1*** | Doublecortin-like kinase 1 | 1.52 | ***GPR137C*** | G protein-coupled receptor 137C | -1.58 |
|  |  | ***FZD3*** | Frizzled homolog 3 | 1.62 |  |  |  |
|  |  | ***ST8SIA2*** | ST8 alpha-N-acetyl-neuraminide alpha-2,8-sialyltransferase 2 | 1.74 | ***OPCML*** | Opioid binding protein/cell adhesion molecule-like | -2.29 |
|  |  |  |  |  | ***PDE5A*** | Phosphodiesterase 5, cGMP-specific | -2.58 |
|  |  |  |  |  | ***SERPINE1*** | Serpin peptidase inhibitor, clade E (nexin, plasminogen activator inhibitor type 1), member 1 | -1.89 |
|  |  |  |  |  | ***PDGFC*** | Platelet derived growth factor C | -1.50 |
| **hsa-miR-125-3p** | -2.49 |  |  |  | ***DGKI*** | Diacylglycerol kinase, iota | -1.52 |
|  |  |  |  |  | ***SGK1*** | Serum/glucocorticoid regulated kinase 1 | -1.56 |
|  |  |  |  |  | ***GPRC5A*** | G protein-coupled receptor, family C, group 5, member A | -1.60 |
|  |  |  |  |  | ***RNF185*** | Ring finger protein 185 | -1.73 |
|  |  |  |  |  | ***MARCH4*** | Membrane-associated ring finger (C3HC4) 4 | -1.86 |
|  |  |  |  |  | ***SLC8A1*** | Solute carrier family 8 (sodium/calcium exchanger), member 1 | -2.16 |
| **hsa-miR-491-5p** | -2.84 |  |  |  | ***SIPA1L3*** | Signal-induced proliferation-associated 1 like 3 | -1.52 |
|  |  |  |  |  | ***SH3RF1*** | SH3 domain containing ring finger 1 | -1.54 |
|  |  |  |  |  | ***MICAL2*** | Microtubule associated monooxygenase, calponin and LIM domain containing 2 | -1.80 |
|  |  |  |  |  | ***ELN*** | Elastin | -2.14 |
|  |  |  |  |  | ***BHLHE40*** | Basic helix-loop-helix family, member e40 | -2.41 |
| **hsa-miR-21-3p** | -3.31 |  |  |  | ***RHOB*** | Ras homolog gene family, member B | -1.92 |
|  |  |  |  |  | ***SMAD7*** | SMAD family member 7 | -2.66 |
|  |  |  |  |  | ***E2F7*** | E2F transcription factor 7 | -1.66 |
|  |  |  |  |  | ***ERCC6*** | Excision repair cross-complementation group 6 | -1.65 |
|  |  |  |  |  | ***NEDD1*** | Neural precursor cell expressed, developmentally down-regulated 1 | -1.57 |
|  |  |  |  |  | ***PPAPDC1A*** | Phosphatidic acid phosphatase type 2 domain containing 1A | -2.36 |
|  |  |  |  |  | ***SSX2IP*** | Synovial sarcoma, X breakpoint 2 interacting protein | -1.79 |
| **hsa-miR-433-3p** | -3.48 | ***CLYBL*** | Citrate lyase beta like | 1.51 | ***EBF3*** | Early B-cell factor 3 | -1.64 |
|  |  |  |  |  | ***GPR137C*** | G protein-coupled receptor 137C | -1.58 |
|  |  |  |  |  | ***MN1*** | Meningioma (disrupted in balanced translocation) 1 | -1.54 |
|  |  |  |  |  | ***E2F7*** | E2F transcription factor 7 | -1.66 |
|  |  |  |  |  | ***ITGA4*** | Integrin, alpha 4 | -2.73 |
|  |  |  |  |  | ***SULF1*** | Sulfatase 1 | -3.11 |
|  |  |  |  |  | ***SFRP2*** | Secreted frizzled-related protein 2 | -14.57 |
| **hsa-miR-27-5p** | -4.32 |  |  |  | ***AFF2*** | AF4/FMR2 family, member 2 | -1.76 |
|  |  |  |  |  | ***CSRNP1*** | Cysteine-serine-rich nuclear protein 1 | -2.80 |
|  |  |  |  |  | ***ENC1*** | Ectodermal-neural cortex 1 (with BTB-like domain) | -1.59 |
|  |  |  |  |  | ***HBEGF*** | Heparin-binding EGF-like growth factor | -2.28 |
|  |  |  |  |  | ***ITGA2*** | Integrin, alpha 2 | -3.05 |
|  |  |  |  |  | ***LHX8*** | LIM homeobox 8 | -1.64 |
|  |  |  |  |  | ***SIK1*** | Salt-inducible kinase 1 | -1.89 |
|  |  |  |  |  | ***STK32B*** | Serine/threonine kinase 32B | -2.83 |
| **hsa-miR-23-5p** | -4.84 | ***FAM49A*** | Family with sequence similarity 49, member A | 2.02 | ***AFF2*** | AF4/FMR2 family, member 2 | -1.76 |
|  |  | ***FKBP5*** | FK506 binding protein 5 | 1.74 | ***ENC1*** | Ectodermal-neural cortex 1 (with BTB-like domain) | -1.59 |
|  |  |  |  |  | ***NPR3*** | Natriuretic peptide receptor C | -3.90 |
|  |  |  |  |  | ***SPOCK1*** | Sparc/osteonectin, cwcv and kazal-like domains proteoglycan 1 | -1.95 |
|  |  |  |  |  | ***MARCH4*** | Membrane-associated ring finger (C3HC4) 4 | -1.86 |
|  |  |  |  |  | ***PCDHB16*** | Protocadherin beta 16 | -2.20 |

^a^DEGs were identified as potential miRNAs targets by querying miRWalk, miRDB, and TargetScan databases.
