# Supplementary material for: Transcriptome-Wide Expression Profiling in Skin Fibroblasts of Patients with Joint Hypermobility Syndrome/Ehlers-Danlos Syndrome Hypermobility Type
Source: PLoS One. 2016 Aug 12;11(8):e0161347. doi: 10.1371/journal.pone.0161347 (PMC4982685; doi:10.1371/journal.pone.0161347)
Supplement: S6 Table — (DOCX) [file pone.0161347.s008.docx]

| **S6 Table. Top canonical pathways perturbed in JHS/EDS-HT skin fibroblasts.** | | | |
| --- | --- | --- | --- |
| **Name** | ***p*-value** | **Ratio^a^** | **DEGs** |
| Osteoclast differentiation | 0.00280619 | 7/123 | ***FHL2, FOS, FOSB, JUNB, NFKBIA****, PPARG,* ***SOCS3*** |
| Arrhythmogenic right ventricular cardiomyopathy | 0.00437592 | 5/69 | ***CDH2, DSP, ITGA2, ITGA4, SLC8A1*** |
| TNF signaling pathway | 0.00527476 | 6/104 | ***FOS, IL6, JUNB, LIF, NFKBIA, SOCS3*** |
| TGF-beta signaling pathway | 0.00609763 | 5/75 | ***ID1, ID3, INHBA, MYC, SMAD7*** |
| MicroRNAs in cancer | 0.00678147 | 10/266 | *FZD3,* ***MET****, MIR21,* *MIR221,* *MIR222,* *MIRLET7F1,* ***MMP16****,* ***MYC****,* ***SPRY2****,* ***ZFPM2*** |
| Jak-STAT signaling pathway | 0.00786915 | 7/150 | ***IL6, IL11, LIF, MYC, PRLR, SOCS3, SPRY2*** |
| PI3K-Akt signaling pathway | 0.0115099 | 11/334 | ***CHRM2****,* ***IL6****,* ***ITGA2****,* ***ITGA4****,* ***MET****,* ***MYC****,* ***NR4A1****,* ***PDGFC****,* ***PRLR****,* ***SGK1****,* ***TEK*** |
| Adherens junction | 0.0216769 | 4/69 | ***MET,***  ***PTPRB, SNAI1, SSX2IP*** |
| Hypertrophic cardiomyopathy | 0.0328022 | 4/79 | ***SLC8A1, IL6, ITGA2, ITGA4*** |
| Metabolism of xenobiotics by cytochrome P450 | 0.047253791 | 4/60 | *AKR1C3, AKR1C2, ADH1C, GSTM5* |
| Calcium signaling pathway | 0.0487054 | 6/175 | ***SLC8A1****,* ***PDE1C****,* ***CHRM2****,* ***SPHK1****,* ***OXTR****,* ***MYLK*** |

^a^: input genes/genes in pathway, the down-regulated genes are reported in bold.
